# The effect of mindfulness-based stress reduction (MBSR) training on serum cortisol levels, depression, stress, and anxiety in type 2 diabetic older adults during the COVID-19 outbreak

**DOI:** 10.25122/jml-2021-0437

**Published:** 2022-12

**Authors:** Ahmad Reza Sayadi, Seyed Hamid Seyed Bagheri, Ali Khodadadi, Reza Jafari Torababadi

**Affiliations:** 1 Department of Psychiatric Nursing, School of Nursing and Midwifery, Rafsanjan University of Medical Sciences, Rafsanjan, Iran; 2 Social Determinants of Health Research Center, Rafsanjan University of Medical Sciences, Rafsanjan, Iran; 3 Department of Pediatric and Neonatal Nursing, School of Nursing and Midwifery, Rafsanjan University of Medical Sciences, Rafsanjan, Iran; 4 Non-Communicable Diseases Research Center, Rafsanjan University of Medical Sciences, Rafsanjan, Iran; 5 Department of Medical Surgical Nursing, School of Nursing and Midwifery, Rafsanjan University of Medical Sciences, Rafsanjan, Iran; 6 Department of Geriatric Nursing, School of Nursing and Midwifery, Rafsanjan University of Medical Sciences, Rafsanjan, Iran

**Keywords:** type 2 diabetes, anxiety, stress, depression, mindfulness, cortisol

## Abstract

Old age is rapidly increasing and is linked to with chronic diseases, especially diabetes. Diabetes is associated with increased anxiety, stress, and depression and, in turn, can increase cortisol secretion. To this end, the present research studied the impact of mindfulness-based stress reduction (MBSR) training on serum cortisol levels, depression, anxiety, and stress in type 2 diabetic (T2DM) older adults during the COVID-19 epidemic. The participants in this interventional work were 56 older adult patients with type 2 diabetes chosen through systematic random sampling and then randomly divided into control and intervention groups containing equal members. In the intervention group, the participants attended eight mindfulness-based stress reduction (MBSR) training sessions. The subjects in the control group received no intervention. Since four patients left the study, only data for 52 patients were collected using the Depression Anxiety Stress Scale (DASS-21) and a demographic and disease information questionnaire. Data were examined with SPSS18 software using the Kolmogorov-Smirnov test, chi-square test, Fisher test, independent samples t-test, and two-way ANOVA; the significance was p<0.05. Statistically significant differences were observed between the mean scores of anxiety, stress, depression, and cortisol levels in the intervention group (p<0.00001) before, directly after, and three months after the intervention. However, no statistically significant difference was observed in the mentioned variables in the control group. The mindfulness-based stress reduction (MBSR) intervention can improve anxiety, depression, stress, and cortisol levels in older adults suffering from T2DM.

## INTRODUCTION

One of the biggest demographic changes in the twentieth century is population aging, which will increase the number of older adults from 600 million in 2000 to 2 billion by 2050. The World Health Organization considers people over 60 to be older adults in third-world countries. The issue of health and its relationship with longevity nowadays has created many challenges. The rate of aging in developing countries is significantly higher compared to developed countries, preventing such countries from adapting to aging consequences. The last 2006 general population and housing census conducted in Iran showed that about 7.3% of the country's population, *i.e*., more than 5 million people, are over 60 years old [1].

Aging changes are associated with health problems and reduced levels of activity. Physical dysfunction increases with age, negatively affecting the ability to maintain independence and increasing the need for help, reducing the quality of life among older adults [2]. Furthermore, the risk of developing chronic diseases increases with age. Lack of mobility also increases the risk of obesity, osteoarthritis, cardiovascular disease, and diabetes [3]. Diabetes is a group of common metabolic disorders associated with high blood sugar. Diabetes is classified based on the pathogenic processes that cause high blood sugar. The two major groups of diabetes are type 1 and type 2, with 5 to 10 percent being the former and the remaining being the latter.

Type 2 diabetes (T2DM) is related to family history, aging, obesity, inactivity, impaired glucose metabolism, and race [4]. It was formerly determined as non-insulin-dependent or adult-onset diabetes. It is commonly diagnosed in people with insulin resistance and a decrease in insulin. There is no need to use insulin at the onset of the disease, and many patients do not require it [4]. Based on the International Diabetes Federation (IDF), the global incidence of diabetes is estimated to increase from 151 million in 2000 to 693 million in 2045. Furthermore, it stated that half of the patients with diabetes were undiagnosed. According to new estimates, the cost of health care due to diabetes follows a heavy social, financial, and health burden worldwide. On the other hand, COVID-19 has significantly increased insulin consumption in diabetic patients [5].

The coronavirus disease (COVID-19) was declared an epidemic on January 30, 2020, by the World Health Organization (WHO) [6]. Iran reported the first case on February 19 [7]. According to the Ministry of Health spokesman, as of July 12, 2021, the number of infected people in Iran was 3373450, and the number of deaths was 85859. In addition, the global number of coronavirus patients was 187632756, and the number of deaths reached 4049071. The mortality rate in the age group below 50 years was less than 1%, and in that over 60, it increased rapidly, reaching 16.9% in the age group 70 to 79 years and 24.4% in the age group over 80 [8]. However, the mortality rate of diabetic cases with COVID-19 was 7.3%, while people with other underlying diseases had lower mortality rates [9]. Diabetic patients suffer from various problems, which are compounded by the association with COVID-19.

Diabetic patients also encounter psychological problems. In recent decades, the psychological aspects of diabetes have attracted the attention of many experts [10]. Previous studies have shown that diabetic patients suffer from symptoms of anxiety, depression, and stress. Diabetes was shown to double the risk of depression and anxiety [11]. More than a quarter of the diabetic population suffers from depression. Depression and stress reduce social interaction and lead to isolation [12]. With the outbreak of COVID-19, older adults may experience anxiety, stress, and withdrawal due to fear of developing the disease [13].

Research has shown that stress plays a key role in managing diabetes. Stress elevates the activity of the hypothalamic-pituitary-adrenal (HPA) axis [14], and cortisol is its final product. Cortisol secretion is lowest in the first half of the night and peaks early in the morning [15]. The relationship between cortisol and eating-related variables is often unclear due to subjective-individual measurements of stress. Although stress measurement tools are related to cortisol levels, the cortisol-stress response varies due to the nature of (social, physical) stressors and how the individual responds to them (psychological response) [16]. As discussed below, different techniques have been used to treat these psychological problems.

One way to overcome psychological problems such as stress in diabetics is to use mindfulness techniques. Mindfulness-based programs (MBP) are concentration programs and consist of sub-disciplines, one of which is mindfulness-based stress reduction [17]. This program is a standard medication program introduced by Kabat-Zinn (1979) as an effective educational solution for individuals with mental health issues and stress. This program aims to help clients learn ways to communicate with life challenges. Mindfulness-based stress reduction is among the psychological techniques described as efficient in treating mental disorders [18, 19].

Mindfulness or presence of mind means "awareness of thoughts, behaviors, emotions, and motivations so that they can be better managed and regulated". Mindfulness means concentrating on senses in a specific way: (1) purposeful presence, (2) being in the present moment, and (3) presence without judgement. This kind of attention leads to increased awareness and acceptance of reality in the present [18–21]. Because older people often take various medications, it is important to identify effective interventions to reduce their need for medication. Studies on different human samples confirmed the efficiency of mindfulness exercises in decreasing anxiety, depression, and stress [22]. There have also been several attempts to use mindfulness-based techniques for older adults [23, 24]. This technique was also used for diabetic patients, but the findings were contradictory [25]. Subsequently, the current study aimed to evaluate the efficiency of mindfulness-based stress reduction (MBSR) training in decreasing blood cortisol levels, anxiety, stress, and depression in adult-onset diabetic patients.

## MATERIAL AND METHODS

The population in this experimental interventional study conducted in 2020 consisted of all cases with adult-onset diabetes who were members of the Diabetes Clinic in Anar County. Following a previous study [26], via the formula:


$$n=2{\left(Z_{1-\frac{\alpha }{2}}+Z_{1-\beta }\right)}^{2}\delta^{2}/d^{2}$$

taking α=0.05 and β=10% and the significant intergroup difference of d=4 and σ=4.04, the minimum essential sample size for each group was estimated at 21 persons. However, taking into account the potential dropout rate, 28 patients were selected for each case and control group based on the inclusion criteria and using systematic random sampling. The inclusion criteria were as follows: being diagnosed with adult-onset diabetes in the medical records of the clinic, not being a confirmed COVID-19 case, not receiving injectable insulin, not having a history of mental ailment, not using psychotropic drugs or cognitive treatments during the study, with no advanced neuropathy, no history of other physical illnesses simultaneously, a history of at least one year of diabetes, no history of coffee, caffeine, smoking, alcohol, and drugs, having middle school education and higher, not attending yoga, meditation, relaxation, special sports activities, lifestyle, and adaptation training courses in the last six months, no hearing and speech problems, no major stress in the last 6 months, not taking corticosteroids, using personal protective equipment, including wearing masks, having a depression score higher than 9, anxiety score higher than 7, and stress score higher than 14 on the Depression Anxiety Stress Scale (DASS21). The exclusion criteria were: refusal to cooperate in the study, positive PCR test, evidence of other endocrine disorders during the study, sudden and drastic change in the patient's general condition associated with changes in blood sugar levels, exacerbation of medical complications of diabetes, and failure to attend three training sessions.

The tools employed to gather the data were a demographic information questionnaire, the Depression Anxiety Stress Scale (DASS-21), and the ELISA test. The demographic information questionnaire evaluated the subjects' demographic characteristics (age, sex, education, and occupation) and information about the disease (the disease duration and family history of diabetes). The DASS-21 was administered to calculate the patients' depression, stress, and anxiety, which was completed by interviewing the patients. The DASS-21 items were scored on a four-point Likert scale (never=0, low=1, moderate=2, and high=3). The reliability and validity of the scale were evaluated by Samani et al. for use in Iran. The test-retest reliability values for the depression, anxiety, and stress subscales were 0.90, 0.76, and 0.77; also, the Cronbach's alpha coefficient for the depression, anxiety, and stress subscales was reported to be 0.91, 0.74, and 0.79, respectively [24].

An ELISA test was used to measure the serum cortisol levels (the Dana ELISA device reader model 3200). After obtaining ethical approval, a permit from Anar County Healthcare Network, and written consent from the participants, 56 diabetic patients were systematically selected from those who met the inclusion criteria. The selected cases were then randomly grouped into intervention and control, each with 28 members. One case in the former and 3 cases in the latter were left out of the research owing to non-attendance and death. Thus, the study was conducted on 52 older adults in the diabetes clinic and health hall of Hazrat Vali Asr Hospital in Anar County, affiliated with Rafsanjan University of Medical Sciences. First, the questionnaires were filled out by the participants in the groups in a session before starting the training intervention. The questionnaires were completed in a hall with a capacity of 200 people with full compliance with health and social distancing protocols. The researchers provided the patients with information on how to answer the questions. Before starting the study, the patients were interviewed and examined by a specialist and a psychologist. The fasting blood samples were obtained at 8:00 am following health and social distancing protocols and transferred to a laboratory to measure serum cortisol levels using the ELISA test.

Then, the members of the intervention and control groups joined two online WhatsApp groups, and the time of the meetings was determined with the participants' agreement. In addition to clinical care and prescribed medication instructions, the subjects in the intervention group joined a mindfulness-based stress reduction (MBSR) intervention training. The training was held in the form of 8 online training sessions. The control group received no intervention except for routine clinical care, including nursing education, blood pressure control, nutrition counseling, visits to an endocrinologist and general practitioner, and prescribed medication instructions. It is noteworthy that the researcher was in contact with the members of both control and intervention groups until the study was finished.

Upon the completion of the intervention, fasting blood samples were taken at 8 am to measure serum cortisol levels following health and social distance protocols. The questionnaires were completed again by participants in both groups on the same day, and the cases in both groups were visited and interviewed by a specialist and a psychologist. In the follow-up phase, three months later, all the steps of the post-intervention phase were repeated. The data collected were codified and analyzed with SPSS18 software employing the chi-square test, Kolmogorov-Smirnov test, independent samples t-test, Fisher test, and two-way repeated-measures analysis of variance (ANOVA). The significance was 0.05 (p<0.05). Furthermore, to comply with ethical values, all mindfulness sessions were conducted for the patients in the control group after completing the study.

## RESULTS

The data for all research variables had a normal distribution. Most participants in both groups were females with a history of diabetes who had a middle school to high school education. In the intervention group, most subjects were retired, and most participants in the control group were housewives. In the intervention group, the mean age of the participants was 68.070±6.613, and that of the participants in the control was 67.52±55.513 years. There was no statistically significant difference concerning demographic characteristics (p>0.05) between groups. The mean duration of diabetes in the intervention group was 8.98±5.65, and in the control group, 8.35±5.19, but no significant intergroup difference was seen (p=0.487). The participants in both groups were taking oral medication, with no significant difference concerning history of smoking, alcohol use, coffee use, and sleep problems (p>0.05) (Table 1).

**Table 1:** Comparing the participants' demographic variables in the groups.

Variable	Categories	Groups		Total Number (%)	X2	df	P-value
		Intervention Number (%)	Control Number (%)				
**Gender**	Male	13 (56.5)	10 (43.5)	23 (100)	0.349	1	0.554*
	Female	14 (48.3)	15 (51.7)	29 (100)			
**Family history of diabetes**	Yes	19 (55.9)	15 (44.1)	34 (100)	0.617	1	0.432*
	No	8 (44.4)	10 (55.6)	18 (100)			
**Education**	Middle school and diploma	22 (51.2)	21 (48.8)	43 (100)	-	-	1**
	Higher education	5 (55.6)	4 (44.4)	9 (100)			
**Occupation**	Self-employed	6 (50)	6 (50)	12 (100)	4.196	2	0.123*
	Housewife	10 (40)	15 (60)	25 (100)			
	Retired	11 (73.3)	4 (26.70	15 (100)			
**Variable**	**Group**	**Number**	**Mean**	**SD**	**t**	**df**	**P-value**
**Age**	Intervention	27	68.0370	6.13613	0.3263	50	0.746**
	Control	25	67.5200	5.20513			
**Duration of diabetes**	Intervention	27	7.98	3.65	0.661	50	0.512***
	Control	25	7.35	3.19			

* – Chi-square test; ** – Fisher exact test; *** – Independent samples t-test.

Moreover, the mean score of anxiety, stress, depression, and serum cortisol levels between the groups before the intervention were not significantly different (p<0.05). However, significant intergroup differences were observed directly after and three months after the intervention (p<0.00001) (Table 2).

**Table 2:** Two-way ANOVA repeated measures analysis of variance results for the research variables.

Variable	Source of changes	Sum of squares	df	Mean square	F	Effect size	Sig.
**Stress**	Time	4345.136	1.662	2614.976	424.251	0.895	<0.00001
	Group-time	3589.444	1.662	2160.186	350.476	0.875	<0.00001
	Error	512.095	83.082	6.164	-	-	-
**Anxiety**	Time	1275.776	2	637.888	146.903	0.746	<0.00001
	Group-time*	811.366	2	405.683	93.427	0.651	<0.00001
	Error	434.224	100	4.342	-	-	-
**Depression**	Time	2472.785	1.427	1733.186	116.755	0.700	<0.00001
	Group-time	14449.913	1.427	1016.250	68.459	0.578	<0.00001
	Error	1058.959	71.336	14.845	-	-	-
**Cortisol**	Time	355.849	2	177.947	10.574	0.281	<0.00001
	Group-time	117.331	2	58.665	6.453	0.114	<0.00001
	Error	909.093	100	9.091	-	-	-

A two-way repeated measures analysis of variance was run to assess the impact of time (change in the mean score of anxiety, stress, depression, and serum cortisol levels during repetitive measurements), group effect (change in the mean score of anxiety, stress, depression, and serum cortisol level during repetitive measurements) and the group-time interaction between (change in the mean score of anxiety, stress, depression, and serum cortisol level over time and considering the impact of groups). The multivariate analysis indicated that the effect of group-time interaction (p<0.00001) and the impact of time was statistically significant (p<0.001). Furthermore, the between-subject effect test showed a statistically significant difference (p<0.00001). Thus, statistical modeling was employed to examine in detail the group-time interaction and, as a result, to examine more accurately the trend of variations in the mean score of anxiety, stress depression, and serum cortisol level in times and groups and the contrast among.

**Figure 1:**
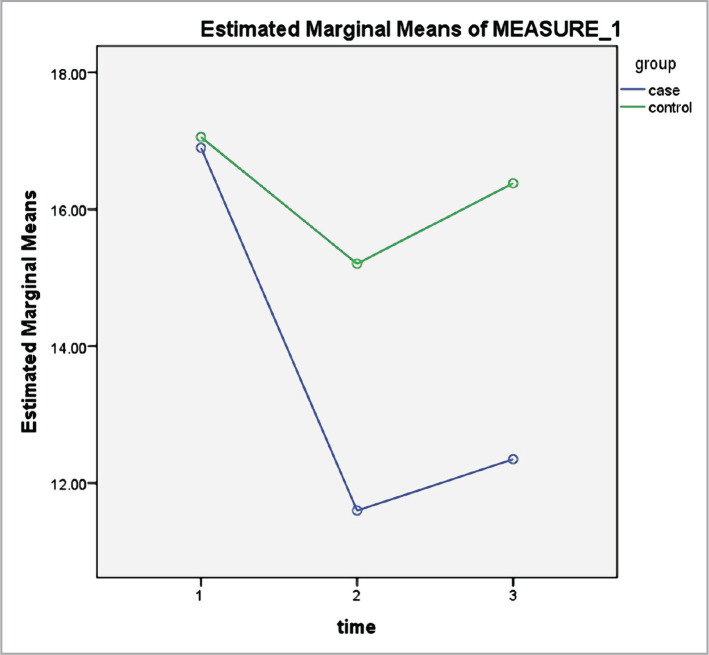
Serum cortisol levels, pre-test, post-test, and three months after the intervention in the case and control groups.

**Figure 2:**
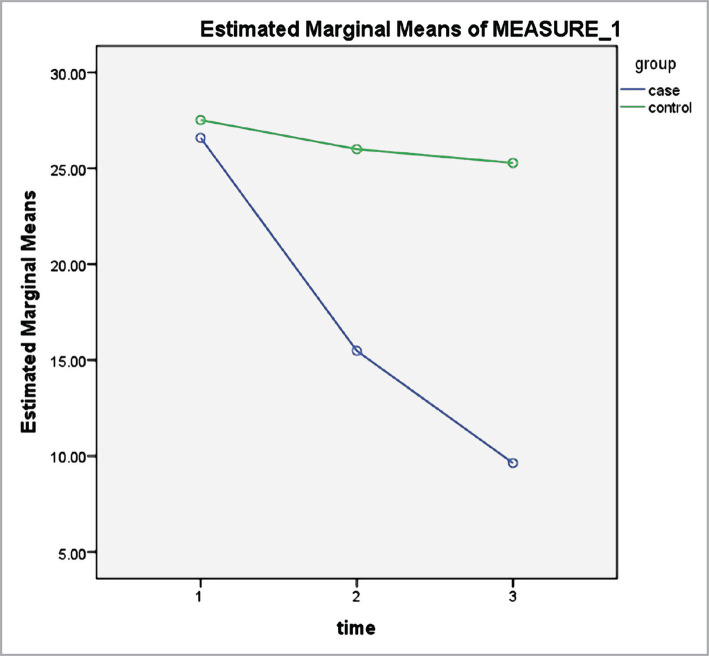
Depression levels, pre-test, post-test, and three months after the intervention in the case and control groups.

**Figure 3:**
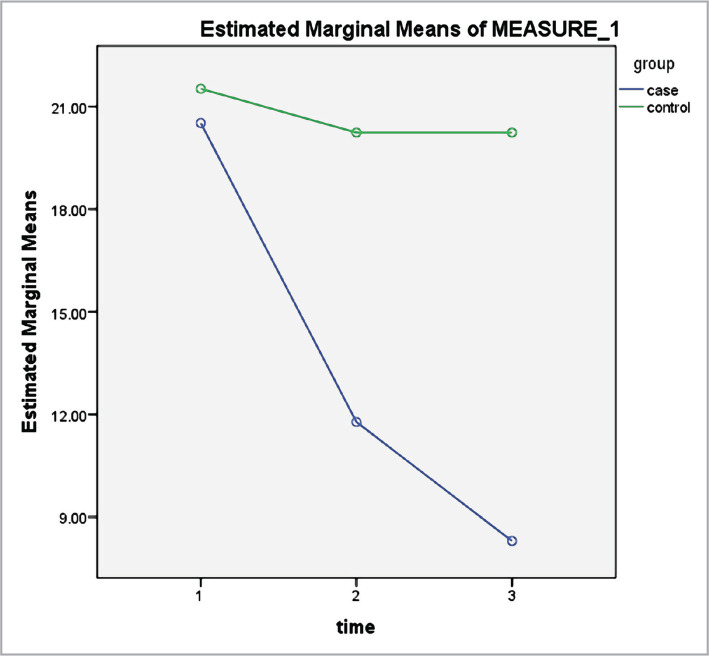
Anxiety levels, pre-test, post-test, and three months after the intervention in the case and control groups.

**Figure 4:**
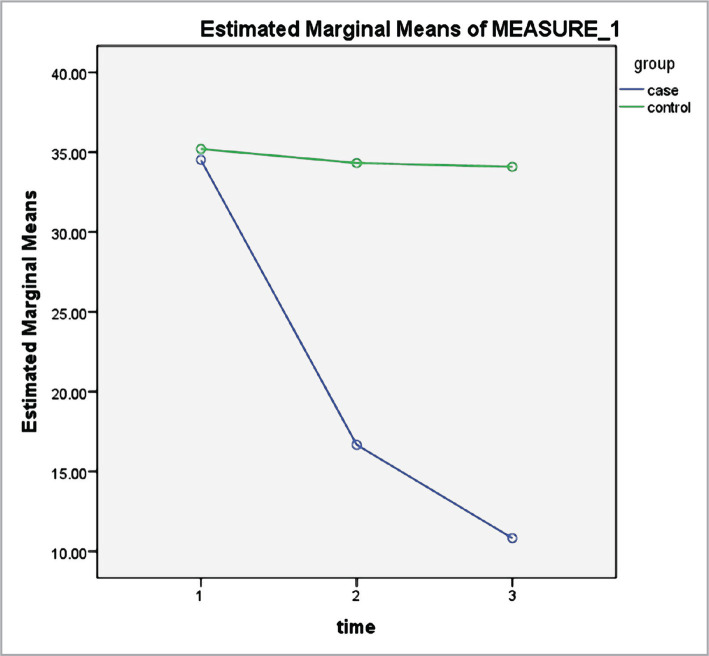
Stress levels, pre-test, post-test, and three months after the intervention in the case and control groups.

According to Table 2, the measurement time significantly impacted the mean scores of serum cortisol (Figure 1), depression (Figure 2), anxiety (Figure 3) and stress (Figure 4) levels (p<0.001). Thus, irrespective of group membership, a significant difference was observed in the mean score of the variables before, directly after, and 3 months after the intervention program (p<0.001). The time-group interaction impact was also significant (p<0.001), indicating the mean score of anxiety, stress, depression, and serum cortisol level for the subjects in the intervention group to be significantly lower compared to the control. This indicates the significant impact of the mindfulness-based intervention in decreasing anxiety, stress, depression, and serum cortisol level in older adult diabetic cases.

According to the results of the intervention group, a significant difference was observed in the stress level in the three stages (p<0.00001); however, no statistically significant difference was observed in the stress score in the control group in all stages (p>0.05). Moreover, based on the assessment of the variations in the anxiety score, a significant difference was observed in the anxiety level in the three stages in the intervention group (p<0.00001); however, there was no statistically significant difference in the anxiety score in the control group in the three stages (p>0.05). According to the analysis of the depression score, a significant difference was observed in the depression levels in the three stages in the intervention group (p<0.00001); however, no statistically significant difference was observed in the depression score in the control group in all stages (p>0.05). Finally, based on the evaluation of the variations in the serum cortisol level, a significant difference was observed in the serum cortisol level in the three stages in the intervention group (p<0.00001); however, no significant difference was observed in their serum cortisol level directly after and three months after the intervention (p=0.830). Moreover, no statistically significant difference was observed in serum cortisol levels reported by the subjects in the three stages in the control group (p>0.05) (Table 3).

**Table 3:** Bonferroni test results for pairwise comparison of mean changes in the research variables.

Variable	Groups	Stage	Standard error±Mean changes	Sig.
**Stress**	case	Pre- & post-intervention	17.85±0.58	<0.00001*
		Pre-intervention & follow-up	23.7±0.74	<0.00001*
		Post-intervention & follow-up	5.85±0.5	<0.00001*
	Control	Pre- & post-intervention	0.88±0.6	0.454
		Pre-intervention & follow-up	1.12±0.77	0.451
		Post-intervention & follow-up	0.24±0.53	1.000
**Anxiety**	case	Pre- & post-intervention	8.78±0.59	<0.00001*
		Pre-intervention & follow-up	12.22±0.62	<0.00001*
		Post-intervention & follow-up	3.48±0.47	<0.00001*
	Control	Pre- & post-intervention	1.28±0.62	0.132
		Pre-intervention & follow-up	1.28±0.65	0.161
		Post-intervention & follow-up	0.000	1.000
**Depression**	case	Pre- & post-intervention	11.11±0.81	<0.00001*
		Pre-intervention & follow-up	16.96±1.12	<0.00001*
		Post-intervention & follow-up	5.85±0.66	<0.00001*
	Control	Pre- & post-intervention	1.52±0.84	0.227
		Pre-intervention & follow-up	2.24±1.17	0.183
		Post-intervention & follow-up	0.72±0.59	0.900
**Cortisol**	case	Pre- & post-intervention	5.3±0.84	<0.00001*
		Pre-intervention & follow-up	4.55±0.99	<0.00001*
		Post-intervention & follow-up	-0.75±0.68	0.830
	Control	Pre- & post-intervention	1.85±0.88	0.118
		Pre-intervention & follow-up	0.67±0.95	1.000
		Post-intervention & follow-up	-1.18±0.71	0.312

## DISCUSSION

According to this study, stress, anxiety, depression, and serum cortisol level were significantly different in the pre-intervention, post-intervention, and after three months for the participants in the case group (p<0.00001). However, no statistically significant difference was observed in stress, anxiety, depression, and serum cortisol level in the three phases in the control group (p>0.05), indicating the efficiency of the mindfulness program in decreasing their level in the case group as compared to the control group. The data in the present work also showed that the measurement time significantly impacted the mean score of all four variable levels. Thus, irrespective of the group membership, a significant difference was seen in the mean score of the variables in the three stages. The time-group interaction impact was also significant, indicating the reduction in the mean score of the variables in the case group to be significantly higher compared to the control group. Similarly, Mir Mehdi et al. (2017) examined the impact of psychological programs with progressive muscle relaxation on depression, anxiety, and stress in type 2 diabetic cases [27]. According to their results, statistically significant differences were observed in the mean score of stress, anxiety, and depression in the case group in the three phases; however, no statistically significant difference was seen in the same variables in the control group in the three phases.

According to the two-way ANOVA results, the mean stress score was significantly different directly and three months after the intervention in the groups; however, there was no significant difference in the stress score prior to the intervention. Mojarad Kahani et al. (2015) examined the efficiency of group psychological training intervention on depression, stress, and anxiety experienced by families of cases with bipolar disorder. The results showed that group psychological training interventions could reduce psychological problems (depression, stress, anxiety) in the families of patients with mood disorders [28]. In a similar study, Bayazi (2012) examined the efficiency of a short cognitive-behavioral intervention on depression, stress, and anxiety in cases with chronic coronary heart illness and showed that the intervention group, as compared with the control, reported a significant reduction in their level of stress after the intervention [26].

Following these findings, it can be argued that the MBSR program increased the coping skills of diabetic patients in the face of stress and thus reduced patients' stress. Furthermore, when diabetic patients are exposed to constant and severe stress induced by the disease, they will be less committed to the treatment programs and are less likely to engage in self-care behaviors, leading to a vicious cycle. However, training interventions, either directly or indirectly, can reduce the levels of stress experienced by patients. It should also be noted that mindfulness reduces stress through mechanisms such as confrontation, acceptance, relaxation, desensitization, changing relationships with thoughts, and emotion regulation [29]. Accordingly, Witek-Janusek et al. (2008) argued that training interventions allow the individual to reduce automatic and habitual responses to stressful experiences, and developing insight and greater acceptance of life-changing events over time can reduce the activation of the stress response system [30].

The present research indicated that considering the group-time interaction, the levels of anxiety reported in the three phases were significantly different for the participants in the intervention group. Nevertheless, no statistically significant difference was observed in the anxiety score in the control group in the three phases. Similarly, Kharatzadeh et al. (2017) examined the efficiency of the MBSR on anxiety, stress, depression, and glycemic control in cases with type 2 diabetes and found that stress and blood glycosylated hemoglobin levels significantly reduced in the intervention group after the intervention as compared with the control. However, no significant difference was seen in the severity of anxiety and depression between the groups after the intervention [31].

In line with the current research, Arab Ghanei et al. (2017) found that MBSR effectively increased assertiveness among anxious students. In addition, a significant difference was observed between anxiety reduction and mindfulness training [32]. In another study, Habibi and Hanasabzadeh (2014) investigated mindfulness-based treatment efficiency on depression, stress, anxiety, and life quality in postmenopausal females. A total of 17 postmenopausal females aged 47 to 60 were chosen in a pre-test-post-test experimental control group design via voluntary sampling and grouped randomly into intervention and control with 9 and 8 participants, respectively. The results of the qualitative analysis indicated a decrease in depression and stress and an increase in their quality of life, but contrary to the present work, it did not significantly impact anxiety [33].

According to the Bonferroni test, a significant difference was observed in depression levels in the three phases for the participants in the intervention group; however, no statistically significant difference was observed in the depression score in any of the phases in the control group. Similarly, Palizgir et al. (2014) compared the effect of psychoeducational and cognitive-behavioral treatments on blood sugar in cases with depression and type 2 diabetes. They indicated cognitive-behavioral group therapy to be more efficient in treating depression in cases with this disease [34]. Accordingly, Hamid (2011) argued that participating in cognitive-behavioral training courses rebuilds one's cognitions and beliefs about diabetes, strengthens positive and hopeful beliefs in patients and reduces depression caused by diabetes as a chronic debilitating disease [35]. Franco et al. showed a significant reduction in depression, anxiety, and worry in the older adults in the intervention group compared to the control group [36]. In another study, Vala et al. examined the mindfulness-based stress reduction program effect on stress, anxiety, depression, self-confidence, as well as hemoglobin A1c in young females with type 2 diabetes and found that anxiety and stress levels were significantly lower and self-confidence was higher in the subjects in the intervention group compared with the control group. Nevertheless, there was no significant difference in the severity of depression between the two groups after the intervention [29]. Moreover, Anderson et al. examined the impact of mindfulness on depression, reducing anger rumination, and increasing concentration and attention in life in 68 adults. Contrary to the present work, the researchers reported insignificant differences between the intervention and the control groups [37–39].

Finally, an evaluation of the variations in the serum cortisol level via repeated measurements in the present study indicated that serum cortisol levels were significantly different in the three phases for the participants in the intervention group. However, there was no significant difference in their serum cortisol level directly after and three months after the intervention. Moreover, the subjects reported no statistically significant difference in serum cortisol levels in the control group during the three phases. Following these findings, it can be argued that mindfulness training significantly lowers blood sugar levels in diabetic cases. On the other hand, the MBSR program, with an effect on the pituitary-hypothalamic axis, leads to a decrease in cortisol and, ultimately, a decrease in depression and blood sugar. Cortisol is an anti-regulatory hormone, and prolonged exposure leads to visceral obesity, insulin resistance, dyslipidemia, and hypertension. This hormone stimulates the sympathetic nervous system, increases inflammatory responses and platelet aggregation, and decreases insulin sensitivity. Therefore, its increase in diabetes is regarded as a risk factor for depression.

Matchim et al. (2011) analyzed 9 articles that addressed the impact of mindfulness on laboratory variables. They concluded that mindfulness has little or no impact on the results of laboratory tests (cortisol, melatonin, and lymphocytes in cancer patients), and it can only affect cognitive variables [36]. This finding partly confirmed our data on cortisol levels in diabetic patients.

The conflicting results found in the literature and the present study can be attributed to training methods and the number and content of training sessions. Mindfulness-based stress reduction training focuses on reducing stress and increasing awareness of one's situation. Leaving the struggle and accepting oneself without judgment is the main concept of MBSR therapy [36].

## CONCLUSION

This research indicated that mindfulness training effectively decreased stress, anxiety, depression, and serum cortisol levels in diabetic older adults. Furthermore, in an interview and survey with the cases in the intervention group after the intervention, they stated that doing mindfulness exercises had a very good effect on them in the difficult conditions caused by the COVID-19 pandemic, which made them feel lonely and anxious. Thus, it can be concluded that mindfulness exercises effectively improved older adults' daily functions and enhanced their quality of life. Consequently, older adults can identify the causes of psychological disorders and stress by implementing mindfulness techniques and trying to reduce them if possible. Otherwise, they can adapt to these problems, which in turn reduces psychological disorders and improves their quality of life.

## Personal thanks

The authors appreciate the Vice-Chancellor for Research and Technology of Rafsanjan University of Medical Sciences, all the patients participating in this research project, and the head and staff of Anarki Laboratory in Anar County.
